# Thyroid cancer and benign thyroid disease in patients with familial adenomatous polyposis (FAP): Memorial Sloan-Kettering Cancer Center (MSKCC) registry experience

**DOI:** 10.1186/1897-4287-9-S1-P24

**Published:** 2011-03-10

**Authors:** Arnold J Markowitz, Gerard Chang, Lisa Cortina, Erin E Salo-Mullen, Jose G Guillem

**Affiliations:** 1 Department of Medicine, Memorial Sloan-Kettering Cancer Center, New York, NY 10065, USA; 2 Department of Surgery, Memorial Sloan-Kettering Cancer Center, New York, NY 10065, USA

## Background

Patients with familial adenomatous polyposis (FAP) are perceived to be at increased risk for developing thyroid cancer. However, screening guidelines for thyroid cancer in FAP patients are not well established. To report the prevalence of thyroid cancer and benign thyroid disease in FAP patients in a single-center hereditary colorectal cancer registry.

## Methods

A retrospective chart review of all FAP patients enrolled in the MKSCC Hereditary Colorectal Cancer Family Registry was performed.

## Results

Sixty six patients with FAP were identified in the registry. This included 30 men and 36 women, with current mean age of 40.6 years. Four patients (6.1%) had a history of thyroid cancer (TC), and underwent thyroidectomy. All TC patients were women, with mean age at cancer diagnosis of 36 years. Three of the 4 known TC diagnoses were papillary thyroid cancer. One patient initially presented with tender thyroid nodule. Another patient with attenuated FAP and a documented APC R332X mutation had a nodule detected on ultrasound. APC mutation testing in the other 3 TC patients is not known. An additional 6 FAP patients (9.1%) were diagnosed with benign thyroid disease, including one with Hashimoto’s thyroiditis, 2 with thyroid nodules, 1 with thyroid cysts, and 2 with multinodular goiter.

## Conclusion

The prevalence of thyroid cancer in our FAP population (6.1%) is increased relative to the general population, and is within the range of that reported by others (1-12%). We are also intrigued by the frequency of benign thyroid disease in this population. We are embarking on a systematic thyroid screening program in our FAP patients.

